# Silicon Enhances Water Use Efficiency by Improving Thermal Responses in *Solanum lycopersicum* L.

**DOI:** 10.3390/plants15213347

**Published:** 2026-10-29

**Authors:** Vinícius Villa e Vila, Gabriel Villela Bedum, Mateus Silva Gelain, Tamara Maria Gomes, Sergio Nascimento Duarte, Patricia Angélica Alves Marques

**Affiliations:** 1 Biosystems Engineering Department, “Luiz de Queiroz” College of Agriculture/ESALQ, University of São Paulo/USP, Padua Dias Avenue 11, Piracicaba 13418-900, SP, Brazil; gabrielbedum@usp.br (G.V.B.); mateus.gelain@usp.br (M.S.G.); snduarte@usp.br (S.N.D.); paamarques@usp.br (P.A.A.M.); 2 Biosystems Engineering Department, Faculty of Animal Science and Food Engineering/FZEA, University of São Paulo/USP, Duque de Caxias 225, Pirassununga 13635-900, SP, Brazil; tamaragomes@usp.br

**Keywords:** canopy temperature, deficit irrigation, thermal camera, water management

## Abstract

Increasing pressure on water resources highlights the need for irrigation strategies that improve water use efficiency (WUE) while maintaining tomato (*Solanum lycopersicum* L.) performance. This study evaluated the effects of silicon (Si) application in the form of silicon dioxide (SiO_2_) on thermal responses assessed using infrared thermography, gas exchange, yield, and WUE under four irrigation levels (40, 60, 80, and 100% crop evapotranspiration, ETc) over two growing seasons. Water deficit progressively impaired physiological performance, with photosynthesis decreasing by up to 20.5% and stomatal conductance by 33.3% under 40% ETc compared with full irrigation. Leaf temperature increased by up to 3.75 °C, while the Crop Water Stress Index (CWSI) reached 0.87. Across all irrigation regimes, the main effect of Si application significantly improved overall plant physiological performance, increasing mean photosynthetic rate by 5.4%, transpiration by up to 16.9%, and leaf relative water content by 3.1%, while decreasing canopy temperature by 0.69–0.84 °C and crop water stress index (CWSI) from 0.60 to 0.62 to 0.44–0.49 across seasons. Si improved WUE, with the highest value (79.07 kg m^−3^) recorded under 40% ETc with Si in the second season, representing a 22.8% increase over the corresponding treatment without Si. Effect-size analysis indicated the largest Si response under moderate water deficit (60% ETc; Hedges’ g = 1.958). Agronomically, 80% ETc optimized the yield–WUE trade-off, cutting water use by 20% and boosting WUE by 14.7% with only an 8.9% yield loss compared to 100% ETc. Furthermore, canopy temperature and CWSI closely reflected plant physiological status across water regimes. These findings demonstrate that thermal imaging can characterize irrigation-related stress responses and that Si application can improve WUE under deficit irrigation, although its benefits depend on water availability and growing conditions.

## 1. Introduction

The global population is projected to approach 10 billion by 2050, increasing the demand for food production and placing additional pressure on water resources [1]. Irrigated agriculture plays a fundamental role in meeting this demand by stabilizing crop production, reducing the effects of rainfall variability, and increasing productivity in regions where precipitation is insufficient to satisfy crop water requirements [2,3]. Irrigated agriculture accounts for approximately 70% of global freshwater withdrawals [4], highlighting the importance of improving irrigation efficiency. However, a large fraction of the water applied through irrigation returns to the hydrological cycle through processes such as evapotranspiration while the excess may infiltrate the soil and contribute to groundwater recharge [5]. Despite representing only about 20% of the global cultivated area, irrigated agriculture produces nearly 40% of the world’s food, fiber, and bioenergy, demonstrating its contribution to agricultural production [4].

The increasing pressure on water resources has intensified the need for irrigation strategies that improve water use efficiency while maintaining crop productivity [6,7,8]. Among these strategies, deficit irrigation has been widely investigated as an approach that supplies less water than the full crop evapotranspiration requirement [9]. By reducing irrigation volumes, deficit irrigation can decrease water use, energy consumption, and production costs, while maintaining acceptable yield levels under appropriate management [2,10,11]. However, its effectiveness depends on the crop’s ability to tolerate water deficit, as reduced soil water availability may impair physiological processes and contribute to yield losses [3,12,13].

Monitoring plant water status is important for evaluating crop responses to deficit irrigation and avoiding water stress levels that impair physiological processes such as photosynthesis, transpiration, and stomatal conductance [14,15]. Among the available approaches, infrared thermography has emerged as a rapid, non-destructive technique for assessing plant water status by detecting changes in canopy temperature [15]. Canopy temperature is closely associated with plant water status because transpiration is one of the main mechanisms regulating leaf energy balance. When stomata closes, transpiration cooling decreases and canopy temperature generally increases. Consequently, canopy temperature has been used as an indirect indicator of crop water status and physiological responses to drought. Recent advances in thermal imaging have made it possible to monitor these responses and provide useful information for irrigation management [16].

In most vegetables, the high water demand results from a combination of physiological and morphological characteristics that intensify water requirements throughout the production cycle [17,18]. Tomato (*Solanum lycopersicum* L.) is one of the most extensively cultivated and consumed vegetable crops worldwide. Global tomato production has increased steadily over the last decades, from approximately 27.6 million t in 1961 to more than 188.5 million t in 2024, with an average yield of 36.81 t ha^−1^ [19]. This increase reflects both the expansion of the cultivated area and improvements in crop productivity, highlighting the growing importance of tomato production in meeting the increasing demand for horticultural products. Despite these advances, tomato remains a crop with high water requirements. Compared with many other food crops, tomato requires approximately 1946 L of water to produce 1000 kcal [20]. This high water demand is associated with its elevated tissue water content, relatively short production cycle, high transpiration rates, and low energy density on a fresh weight basis [21,22]. In tomato, severe water deficit may impair physiological processes and reduce fruit yield and quality [23]. Therefore, combining moderate deficit irrigation with other management strategies that enhance plant tolerance to water stress may contribute to maintaining crop performance while reducing irrigation water use.

To further enhance irrigation management efficiency, the use of bioinputs has gained prominence in agriculture [24,25]. Bioinputs contribute to plant growth and development and strengthen stress responses by interacting with physical, chemical, and biological processes in both soil and plant [26,27]. Among the main categories of bioinputs are microbial inoculants, biological control agents, biofertilizers and biostimulants [26]. These products can be applied to plants, seeds, or soil with the aim of stimulating specific physiological processes that enhance plant growth, stress tolerance, and nutrient use efficiency [28].

Silicon (Si) has been studied as a beneficial element that may reduce the effects of abiotic stress in several crops [29]. Although it is not considered an essential nutrient for most species, Si application has been associated with changes in plant water relations, antioxidant metabolism, membrane stability, and photosynthetic performance under water stress conditions. However, the responses vary according to species, genotype, environmental conditions, Si source, and application method, and the physiological mechanisms [30,31]. Therefore, the beneficial effects attributed to Si are not the result of a single physiological response, but rather the interaction of multiple mechanisms involved in plant adaptation to water-limited environments [31,32]. In addition to its effects on plant water relations and photosynthetic performance, Si may also influence plant thermal responses under water deficit [25]. Because canopy temperature reflects the balance between energy absorption and transpiration cooling, changes in thermal behavior may provide insight into the capacity of plants to maintain physiological activity under reduced water availability [33].

Although the beneficial effects of Si on drought tolerance have been reported in several crops, most studies have focused on growth, yield, gas exchange, and biochemical responses [23,25,30,31]. In contrast, less attention has been given to plant thermal responses, despite canopy temperature being closely associated with transpiration, stomatal regulation, and plant water status. Because changes in canopy temperature integrate several physiological processes affected by water deficit, evaluating plant thermal behavior may provide additional insight into the mechanisms through which Si influences crop performance under limited water availability. Therefore, integrating quantitative thermal imaging assessments with physiological measurements provides a more comprehensive understanding of Si-mediated responses in tomato plants under deficit irrigation. This approach allows the effects of Si on canopy temperature, plant water status, and gas exchange to be evaluated simultaneously, providing quantitative evidence of its contribution to thermal regulation and physiological performance under different levels of water availability.

The objective of this study was to investigate the role of Si in improving water use efficiency and crop yield in tomato plants under deficit irrigation, focusing on its influence on thermal responses and their link to physiological water status and gas exchange over two tomato seasons.

## 2. Results

### 2.1. Crop Water Demand

**Figure 1:**
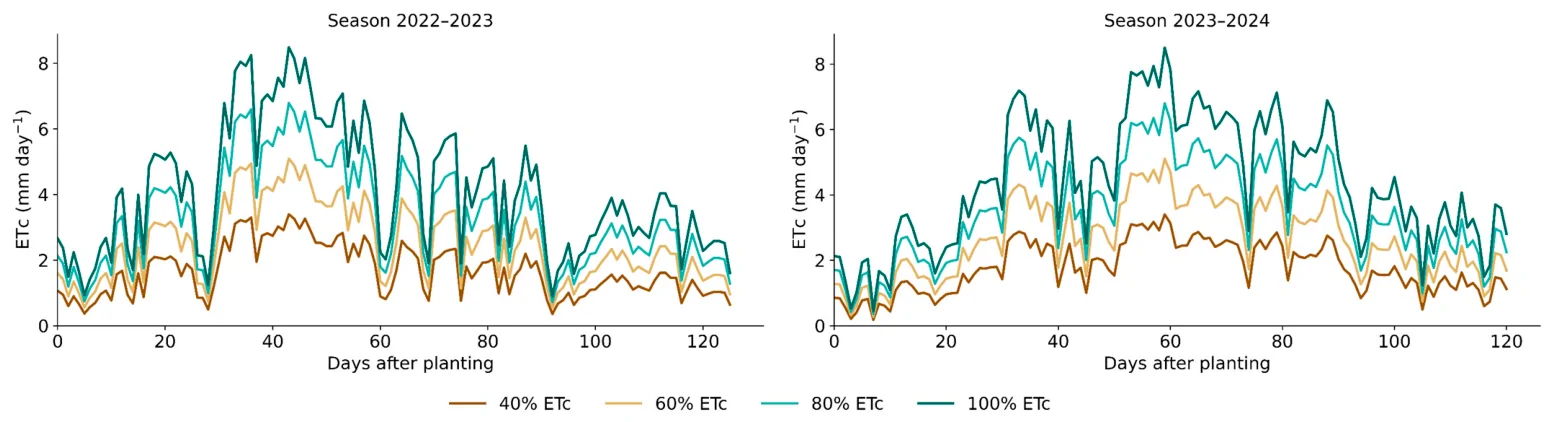
Crop evapotranspiration (ETc) under different irrigation replacement levels during tomato cultivation in the 2022–2023 and 2023–2024 growing seasons.

The variation in crop evapotranspiration (ETc) and the corresponding irrigation replacement levels during the two tomato growing seasons are shown in Figure 1. The first growing cycle lasted 125 days, whereas the second was completed in 120 days. In the 2022–2023 growing season, the cumulative water demand under 100% ETc was 521.72 mm, resulting in cumulative irrigation depths of 417.38, 313.03, and 208.69 mm for the 80, 60, and 40% ETc treatments, respectively. Under 100% ETc, the mean daily water demand was 4.14 mm d^−1^, ranging from a minimum of 0.90 mm d^−1^ at 92 days after planting (DAP) to a maximum of 8.49 mm d^−1^ at 43 DAP. In the 2023–2024 growing season, cumulative water demand under 100% ETc reached 533.39 mm, with cumulative irrigation depths of 426.71, 320.03, and 213.35 mm for the 80, 60, and 40% ETc treatments, respectively. For the 100% ETc treatment, mean daily water demand was 4.41 mm d^−1^, with a minimum of 0.45 mm d^−1^ at 7 DAP and a maximum of 8.50 mm d^−1^ at 59 DAP. In both growing seasons, the highest ETc values occurred predominantly during the middle portion of the crop cycle, followed by a decrease in water demand during the final growth stages.

### 2.2. Silicon Concentration

**Figure 2:**
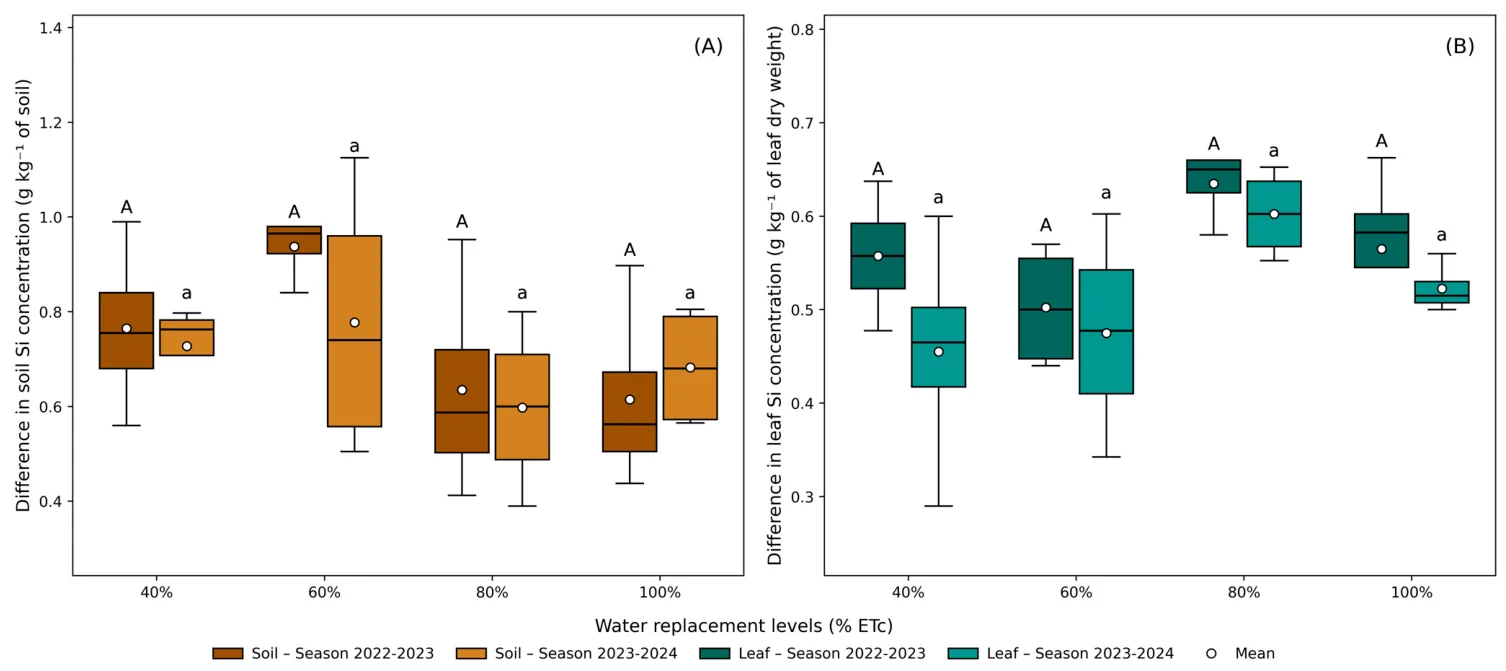
Differences in Si concentration in soil (**A**) and tomato leaves (**B**) under different irrigation water replacement levels during the 2022–2023 and 2023–2024 growing seasons. Differences were calculated as the concentration in the treatment with Si application (+Si) minus that in the treatment without Si application (−Si). Different letters indicate significant differences according to Tukey’s test (*p* < 0.05) within each growing season. Uppercase letters correspond to the 2022–2023 growing season, whereas lowercase letters correspond to the 2023–2024 growing season. ETc: Crop evapotranspiration.

Figure 2 shows the differences in Si concentration in tomato leaves and soil at the end of the two growing seasons, calculated by subtracting the values obtained in treatments without Si application from those in treatments with Si application. Similar patterns were observed in the 2022–2023 and 2023–2024 growing seasons. Si application increased the concentration of this element in both evaluated attributes, with soil values averaging 23.45% and 26.21% higher than those observed in leaves in the first and second growing seasons, respectively. No significant differences were observed among ETc replacement levels. However, under full irrigation and moderate water deficit, corresponding to 100 and 80% ETc, respectively, soil Si concentration decreased by an average of 26.58% in the 2022–2023 growing season and 14.95% in 2023–2024 compared with the mean of the other treatments. In leaves, the opposite pattern was observed, with average increases of 11.67% and 17.33% under these same irrigation levels in the 2022–2023 and 2023–2024 growing seasons, respectively.

### 2.3. Crop Physiological Responses

Table 1 presents the physiological responses of tomato plants to the different irrigation replacement levels and Si application across the two cropping cycles. Irrigation level significantly affected photosynthesis, stomatal conductance, transpiration, and relative water content (RWC) in both seasons (*p* < 0.001). Overall, all physiological parameters increased with increasing water availability, with the highest values generally observed under 100% ETc. In the 2022–2023 season, photosynthesis increased from 24.25 μmol m^−2^ s^−1^ at 40% ETc to 30.50 μmol m^−2^ s^−1^ at 100% ETc. Similarly, stomatal conductance increased from 0.16 to 0.22 mol m^−2^ s^−1^, while transpiration increased from 7.57 to 9.42 mmol m^−2^ s^−1^ and RWC increased from 73.20 to 83.84%. In the 2023–2024 cycle, the same progressive response was observed, with photosynthesis increasing from 24.95 to 30.99 μmol m^−2^ s^−1^, stomatal conductance from 0.16 to 0.24 mol m^−2^ s^−1^, transpiration from 7.42 to 8.85 mmol m^−2^ s^−1^, and RWC from 71.68 to 85.62% between 40 and 100% ETc.

Reducing irrigation levels progressively impaired physiological performance in both growing seasons. Compared with 100% ETc, the 40% ETc treatment reduced photosynthesis by 19.5–20.5%, stomatal conductance by 27.3–33.3%, transpiration by 16.2–19.6%, and RWC by 12.7–16.3%, whereas less pronounced reductions were observed under 60 and 80% ETc. In contrast, Si application increased photosynthesis by 5.4% in both seasons, transpiration by 4.7% and 16.9%, and RWC by 3.1% and 3.0% in 2022–2023 and 2023–2024, respectively. Stomatal conductance increased by 10.5% with Si application only in 2023–2024, with no significant effect observed in the first season.

The ETc × Si interaction was not significant for photosynthesis, stomatal conductance, or RWC in either cropping cycle, indicating that the responses of these variables to Si application were consistent across irrigation levels. In contrast, the interaction was significant for transpiration in both cycles, indicating that the effect of Si on transpiration varied according to water availability. In the first cycle, considering the same irrigation levels, Si increased transpiration by 11.45, 3.64, 5.26, and 0.11% at 40, 60, 80, and 100% ETc, respectively, with the largest increase occurring under the most severe water deficit. In the second cycle, the corresponding increases were 11.11, 21.00, 16.88, and 18.11%, respectively. Thus, although Si increased transpiration across irrigation levels, the magnitude of this response varied with irrigation availability, particularly in the second cycle. Notably, at 80% ETc + Si, transpiration was statistically similar to that observed at 100% ETc − Si in the first cycle and even higher in the second cycle. This indicates that Si application under moderate water deficit allowed tomato plants to maintain transpiration at levels comparable to or greater than those observed under full irrigation without Si.

### 2.4. Crop Thermal Responses

**Figure 3:**
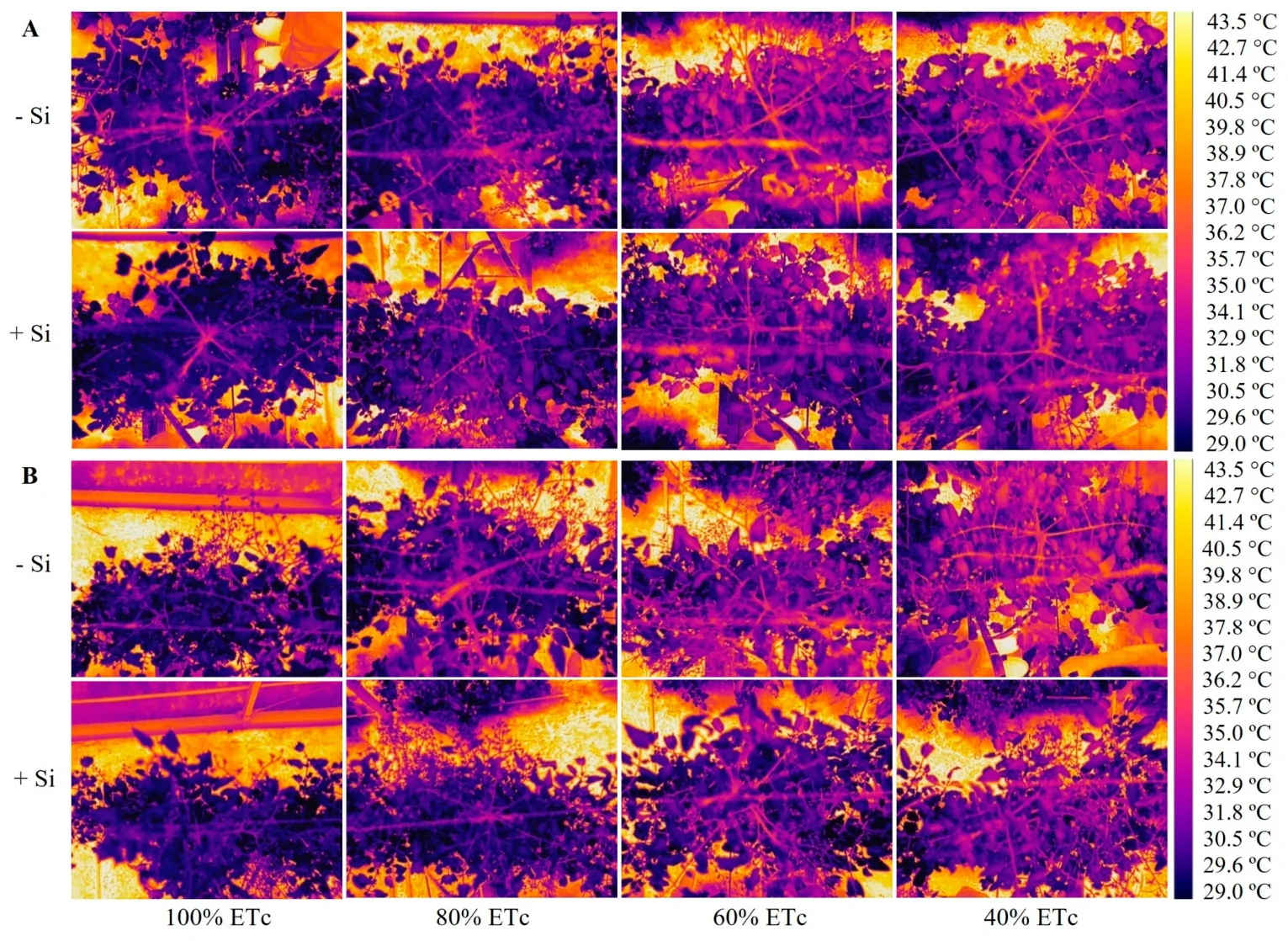
Thermal images obtained under different water replacement scenarios and silicon application treatments. (**A**) 2022–2023 and (**B**) 2023–2024 cropping cycles. Si: silicon; ETc: crop evapotranspiration.

Table 2 presents the thermal responses of tomato plants under different irrigation replacement levels and Si application. Irrigation level significantly affected leaf temperature, ΔT, and CWSI in both cropping cycles (*p* < 0.001). A clear gradient in plant thermal response was observed as water availability decrease (Figure 3). Leaf temperature increased progressively with decreasing irrigation, whereas both ΔT and CWSI increased under greater water restriction. In the 2022–2023 cycle, leaf temperature increased from 31.47 °C at 100% ETc to 34.81 °C at 40% ETc, corresponding to an increase of 3.34 °C. During the same period, ΔT increased from −2.39 to 0.96 °C and CWSI from 0.17 to 0.87, considering 100% ETc to 40% ETc. A similar response occurred in 2023–2024, when leaf temperature increased from 31.42 °C under 100% ETc to 35.17 °C under 40% ETc, while ΔT increased from −1.02 to 2.73 °C and CWSI from 0.31 to 0.80. The intermediate irrigation treatments showed a progressive transition between the fully irrigated and severely water-limited conditions.

Si application significantly reduced leaf temperature, ΔT, and CWSI in both cropping cycles. In 2022–2023, Si application reduced leaf temperature from 33.49 to 32.80 °C, ΔT from −0.35 to −1.05 °C, and CWSI from 0.60 to 0.44. In 2023–2024, the corresponding reductions were from 33.75 to 32.91 °C, from 1.31 to 0.47 °C, and from 0.62 to 0.49, respectively. Thus, plants receiving Si maintained lower canopy temperatures and lower thermal stress indicators than plants without Si, regardless of cropping cycle.

The ETc × Si interaction was significant only for CWSI in the 2022–2023 cycle (*p* < 0.05). In the first season, Si application reduced CWSI particularly under moderate and severe water restriction, with values decreasing from 0.80 to 0.50 at 60% ETc and from 0.44 to 0.33 at 80% ETc. Under 100% ETc, CWSI reached the lowest value with Si application (0.08), compared with 0.26 without Si. In the 2023–2024 season, although the interaction was not significant, the treatment means showed a consistent reduction in CWSI with increasing irrigation and, in general, with Si application. The lowest values were observed under 100% ETc, reaching 0.33 without Si and 0.28 with Si. Overall, the thermal parameters responded consistently to water availability, with greater irrigation reducing leaf temperature, ΔT, and CWSI, while Si application was associated with lower thermal stress indicators across both cropping cycles. These results demonstrate that the thermal indicators consistently differentiated the irrigation treatments according to water availability, with clear visual differences observed in the thermal images (Figure 3).

### 2.5. Crop Yield and Water Use Efficiency

**Figure 4:**
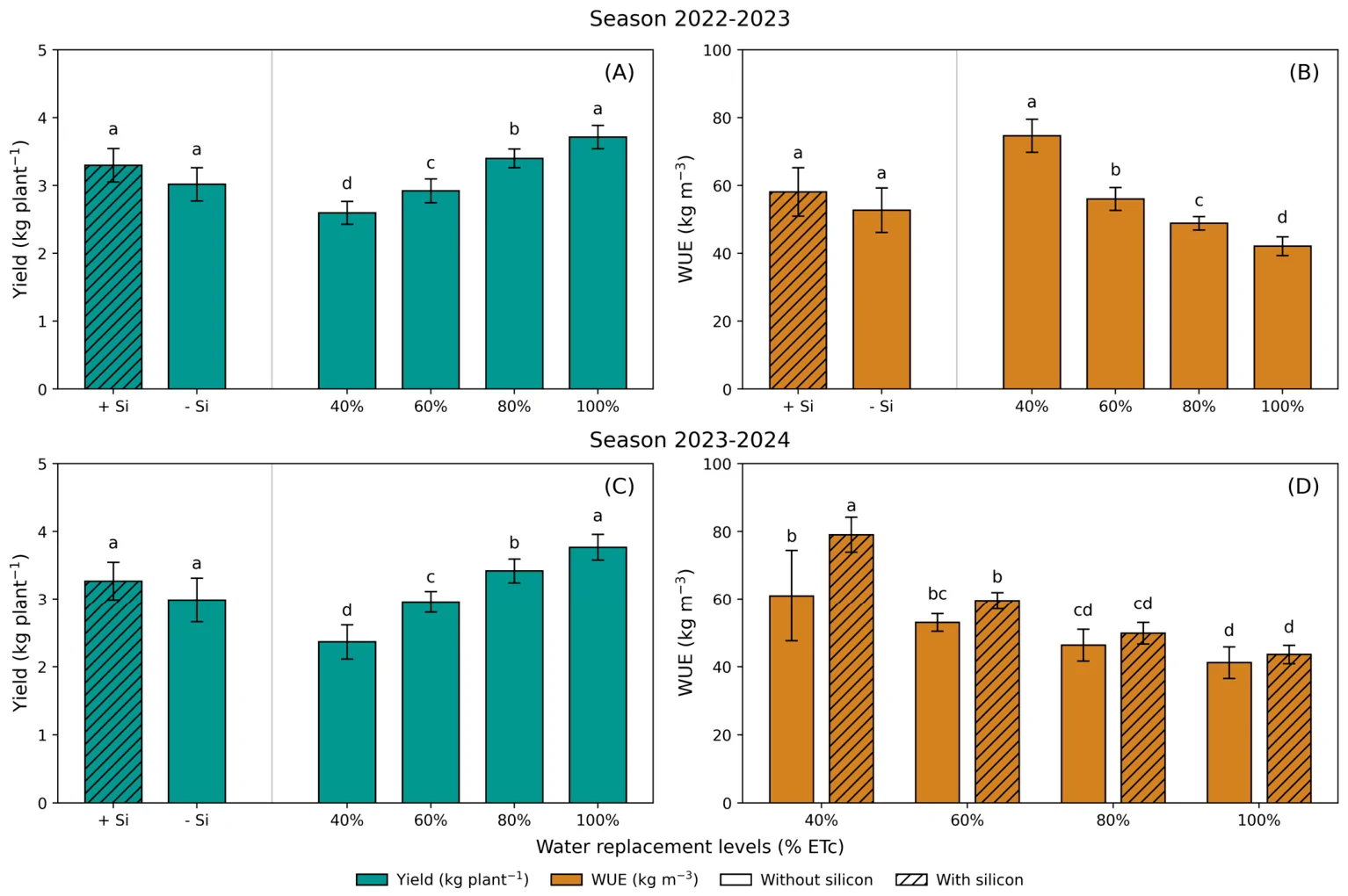
Tomato yield (kg plant^−1^) and water use efficiency (WUE; kg m^−3^) under different water replacement levels and silicon application. (**A**) Yield in the 2022–2023 season; (**B**) WUE in the 2022–2023 season; (**C**) yield in the 2023–2024 season; (**D**) WUE in the 2023–2024 season. Different letters within the same panel indicate statistically significant differences by Tukey’s test at 5% significance. (**D**) presents the mean values for the significant interaction between irrigation levels and Si application, whereas no significant interaction was observed in (**A**–**C**). ETc: crop evapotranspiration; Si: silicon; WUE: water use efficiency.

Figure 4 shows yield and water use efficiency (WUE) as a function of irrigation replacement levels and Si application during the 2022–2023 and 2023–2024 growing seasons. Yield increased with increasing ETc replacement in both seasons. In the first season, mean yield increased from 2.60 kg plant^−1^ at 40% ETc to 3.71 kg plant^−1^ at 100% ETc, corresponding to an increase of 30.06%. In the 2023–2024 season, yield ranged from 2.36 kg plant^−1^ at 40% ETc to 3.77 kg plant^−1^ at 100% ETc, representing an increase of 37.26%. In both growing seasons, irrigation replacement levels differed significantly (*p* < 0.05), with yield increasing progressively as ETc replacement increased. When the effect of Si application was evaluated separately, mean yield was numerically higher in treatments receiving Si in both seasons; however, no significant differences in yield were observed between treatments with and without Si application (*p* > 0.05). In 2022–2023, yield increased by 8.51% with Si application, whereas an increase of 8.57% was observed in the second season. However, no significant interaction was detected between Si application and irrigation replacement levels, indicating that the yield response to Si was independent of the ETc replacement level.

In contrast, WUE exhibited a pattern opposite to that of yield in response to irrigation replacement levels, with higher values under greater water deficit. In the 2022–2023 growing season, WUE decreased from 74.67 kg m^−3^ at 40% ETc to 42.14 kg m^−3^ at 100% ETc, corresponding to a reduction of 43.57%. Si application also increased WUE during this season, with mean values of 58.13 kg m^−3^ in the presence of Si and 52.72 kg m^−3^ in its absence, representing an increase of 9.31%. Significant differences were observed for both factors (*p* < 0.05). However, no significant interaction was detected between irrigation replacement levels and Si application during the first growing season.

In contrast, the 2023–2024 growing season showed a significant interaction between irrigation replacement levels and Si application for WUE. The highest value was observed under severe water deficit with Si application, reaching 79.07 kg m^−3^. The corresponding treatment without Si yielded 61.01 kg m^−3^, representing a difference of 22.84%. These treatments differed significantly, whereas the treatment with Si was statistically equivalent to those receiving 60% ETc. At this irrigation level, WUE was 10.98% higher with Si application, reaching 59.54 kg m^−3^, compared with 53.00 kg m^−3^ without Si. The 80 and 100% ETc levels did not differ significantly (*p* > 0.05). Nevertheless, Si application resulted in slight increases in WUE of 7.00% and 5.46% at 80 and 100% ETc, respectively.

### 2.6. Multivariate Analysis and Response Magnitude

**Figure 5:**
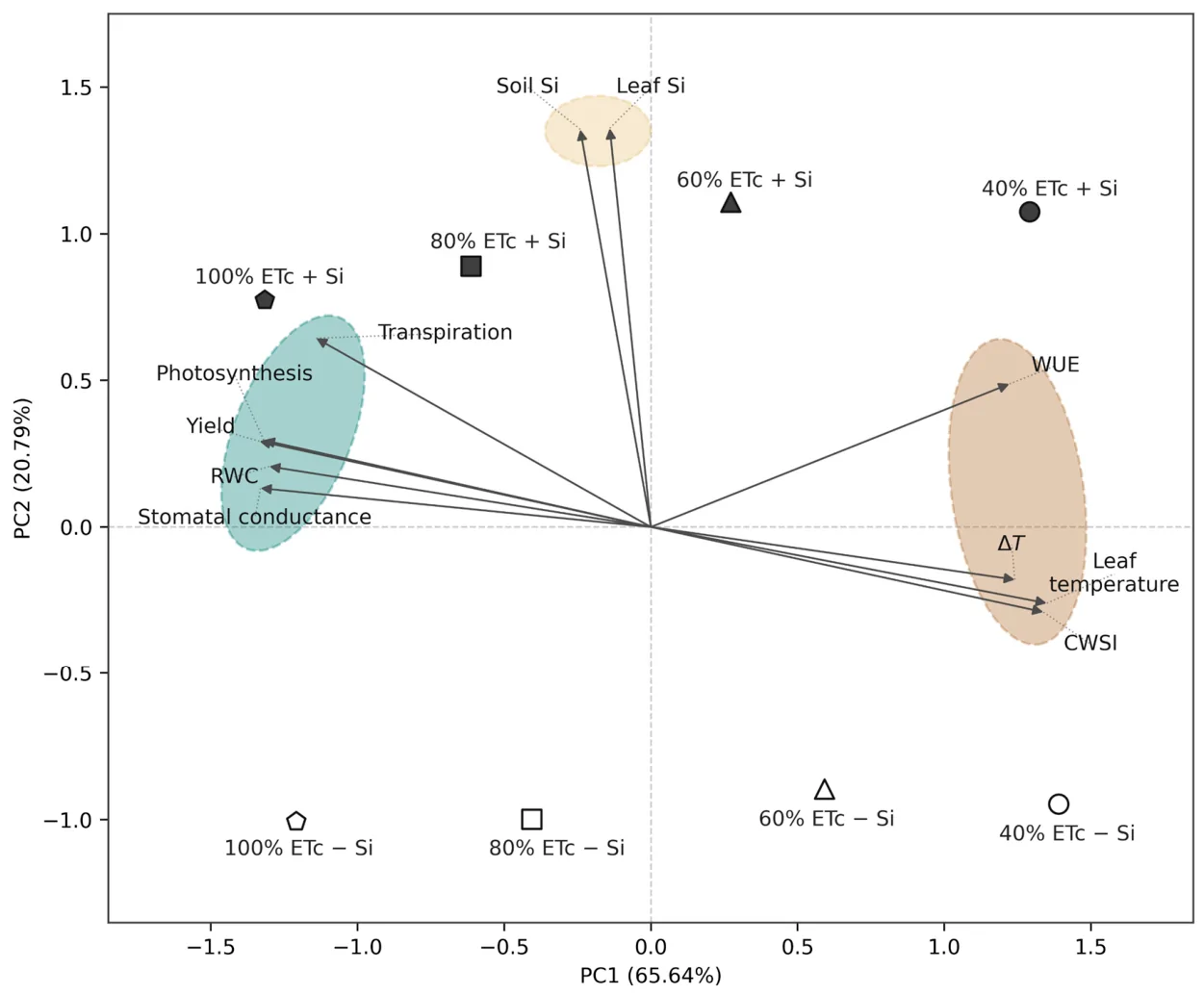
Principal component analysis for all the variables analyzed in the tomato crop, considering the two crop cycles. ETc: crop evapotranspiration; RWC: relative water content; CWSI: crop water stress index; WUE: water use efficiency; Si: silicon.

Principal component analysis explained 84.75% of the total variance, distributed between PC1 (65.64%) and PC2 (20.79%) (Figure 5). In PC1, variables related to plant water status, gas exchange, and yield exhibited loadings in the opposite direction to those of canopy thermal variables and water use efficiency. The variables with the highest contributions to this component included canopy temperature (0.928), stomatal conductance (−0.926), yield (−0.925), CWSI (0.924), and photosynthesis (−0.920). PC2 was predominantly associated with Si concentrations in leaves (0.943) and soil (0.940).

The distribution of treatments along PC1 revealed a gradient associated with the intensity of water restriction. Treatments receiving 100 and 80% ETc were positioned close to the vectors representing physiological and yield-related variables, whereas reducing irrigation replacement to 60 and 40% ETc resulted in progressively more positive scores and closer alignment with the vectors for canopy temperature, CWSI, ΔT, and WUE. Along PC2, treatments receiving Si application exhibited positive scores and were aligned with the vectors for leaf and soil Si concentrations, whereas treatments without Si remained in the negative half-axis. At the same irrigation replacement level, Si-treated plants also exhibited lower PC1 scores and were positioned closer to the physiological and yield-related variables than their respective treatments without Si, particularly under intermediate levels of water restriction.

**Figure 6:**
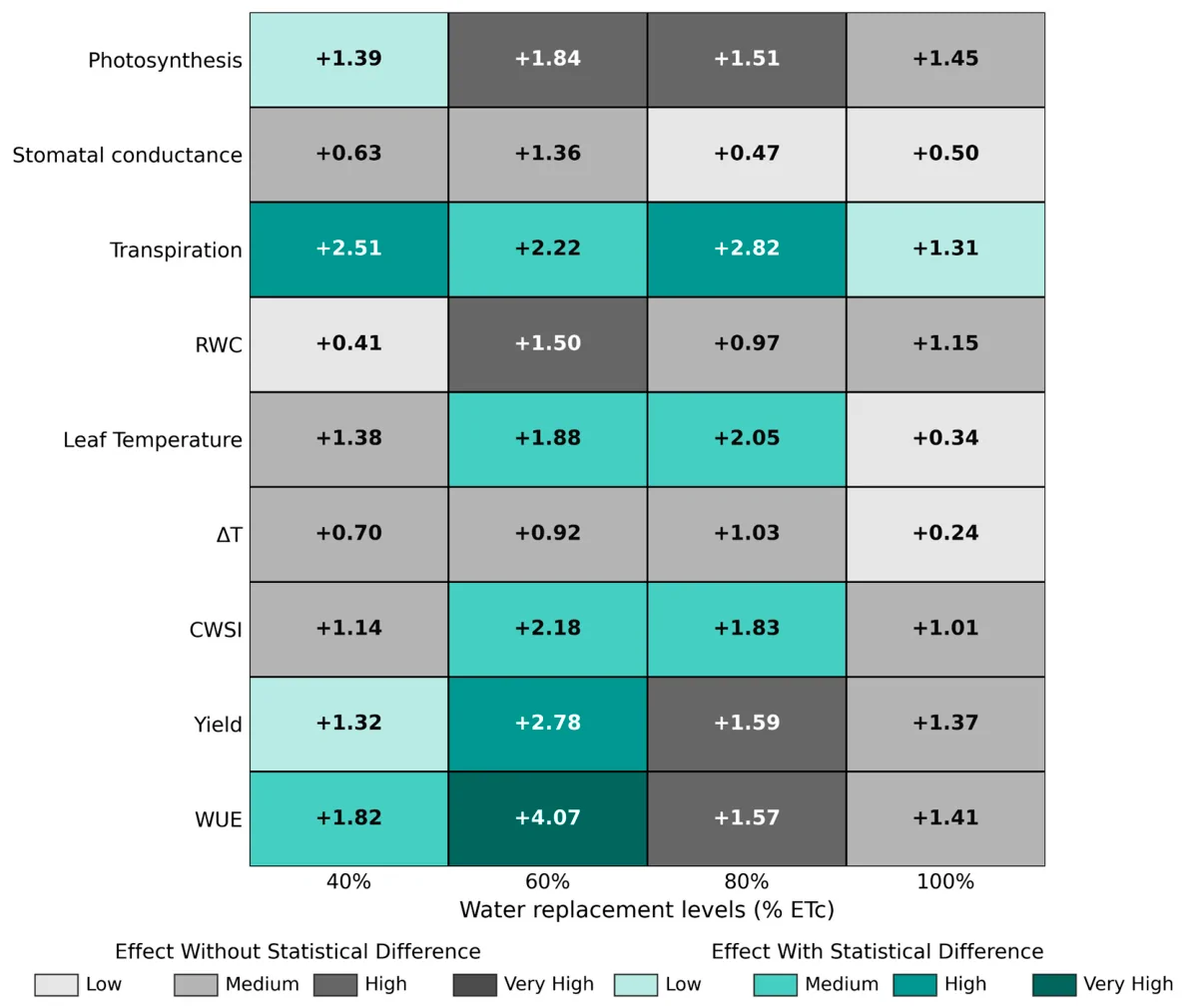
Magnitude and direction of the silicon application effect on physiological, thermal, and yield-related variables of tomato plants under different irrigation replacement levels, considering the 2022–2023 and 2023–2024 growing seasons jointly. Green cells indicate significant beneficial effects, based on Tukey’s test at the 5% significance level and |g| ≥ 0.50. Gray cells indicate non-significant effects or effects with |g| < 0.50. ETc: crop evapotranspiration; RWC: relative water content; CWSI: crop water stress index; WUE: water use efficiency.

Figure 6 illustrates the magnitude of the response to Si application for physiological, thermal, and yield-related variables at different ETc replacement levels. Overall, Si application had beneficial effects on plants, with greater responses under 40 to 80% ETc, particularly at 60% ETc, where Si produced the largest mean effect across variables (mean Hedges’ g = 1.958). In contrast, under full irrigation, responses without statistically significant differences predominated (*p* > 0.05), and the magnitude of the Si effect was lower, with a mean Hedges’ g of 0.898. This pattern indicates that the benefits of Si became more evident as plants were subjected to water restriction, although response magnitude varied among physiological, thermal, and yield-related processes. Among the physiological variables, transpiration showed the most consistent response, with beneficial effects at all irrigation replacement levels and greater effect magnitudes at 40 and 80% ETc. In contrast, photosynthesis, stomatal conductance, and RWC predominantly showed responses without statistically significant differences, indicating that the effects of Si were not generalized across all physiological variables.

Thermal responses were more sensitive to intermediate levels of water restriction. Leaf temperature and CWSI showed beneficial effects mainly at 60 and 80% ETc, whereas ΔT did not exhibit a statistically supported response at any irrigation replacement level. This pattern suggests that the influence of Si on plant thermal status was more pronounced under moderate water deficit than under full irrigation or more severe water restriction. Agronomic variables also showed greater responses under water restriction. Yield responded positively to Si application at 40 and 60% ETc, while WUE showed beneficial effects at these same levels, with the largest effect magnitude observed at 60% ETc. At 80 and 100% ETc, the effects on yield and WUE were not statistically supported. Considering all evaluated variables jointly, 60% ETc concentrated the largest number of statistically supported beneficial responses to Si application and exhibited the highest mean effect magnitude among the irrigation replacement levels.

### 2.7. Trade-Off Between Yield and Wue Under Thermal Stress

**Figure 7:**
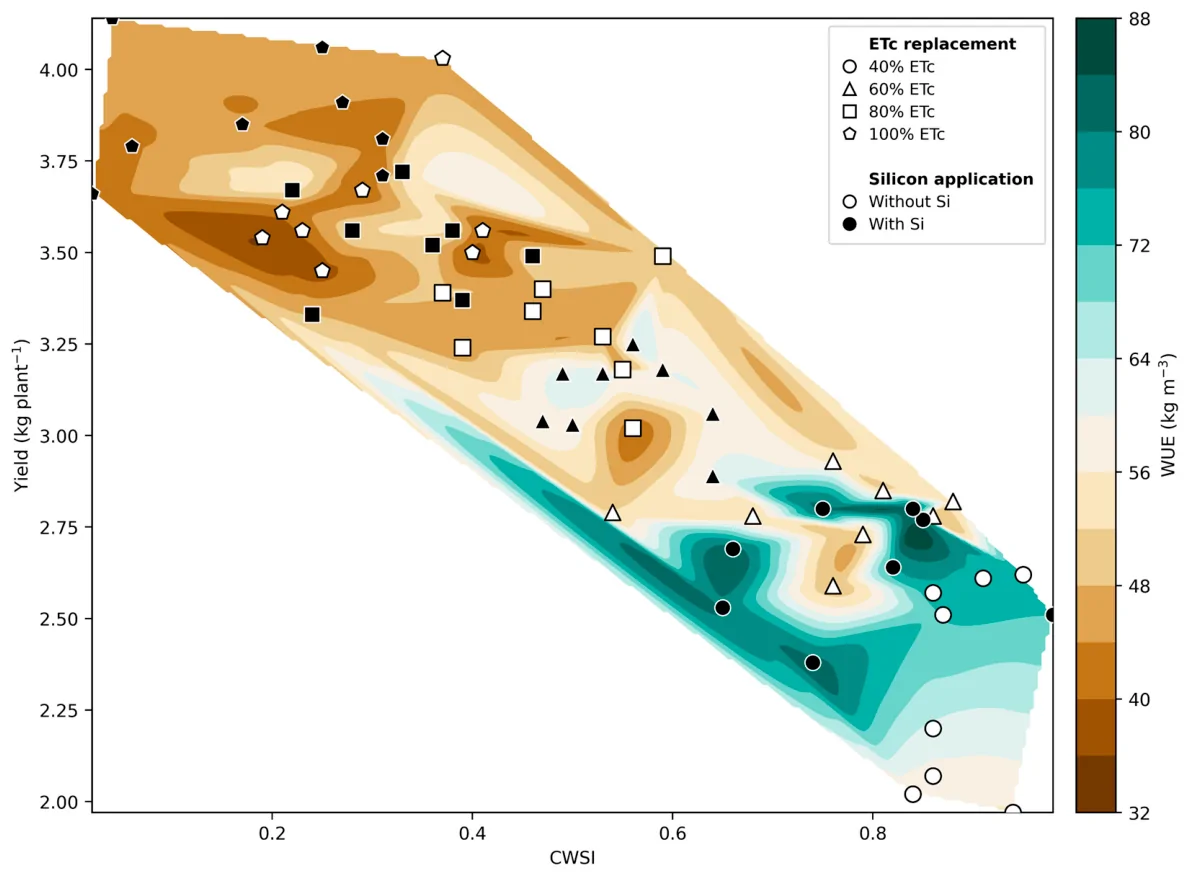
Relationship between crop water stress index (CWSI), tomato yield, and water use efficiency (WUE) under different irrigation replacement levels and silicon application. The color gradient represents interpolated WUE values (kg m^−3^), while symbols indicate irrigation replacement levels (40, 60, 80, and 100% ETc) and symbol filling indicates treatments without or with silicon application.

The integration of CWSI, yield, and WUE revealed a response gradient associated with decreasing irrigation replacement levels (Figure 7). Under 100% ETc, observations showed a mean CWSI of 0.24 and a yield of 3.74 kg plant^−1^, associated with the lowest WUE (42.26 kg m^−3^). Irrigation at 80% ETc resulted in a CWSI of 0.41 and a WUE of 48.48 kg m^−3^, while yield remained at 3.41 kg plant^−1^. Relative to full irrigation, this regime reduced water application by 20%, with an 8.9% decrease in yield and a 14.7% increase in WUE. Further intensification of water deficit widened the differences between water use efficiency and yield performance. At 60% ETc, CWSI reached 0.66 and WUE reached 56.15 kg m^−3^, while yield was 21.4% lower than that observed at 100% ETc. At 40% ETc, WUE reached 72.35 kg m^−3^ but was associated with a CWSI of 0.84 and a yield of 2.48 kg plant^−1^, corresponding to a 33.7% reduction relative to full irrigation. Thus, the greatest gains in WUE occurred alongside increases in CWSI and larger yield reductions, indicating that maximizing water use efficiency did not coincide with maintaining yield performance.

When irrigation levels were considered jointly, Si application was associated with higher mean yield and WUE and a lower CWSI. Plants receiving Si had a yield of 3.28 kg plant^−1^, a WUE of 58.07 kg m^−3^, and a CWSI of 0.46, whereas plants without Si had corresponding values of 3.00 kg plant^−1^, 51.55 kg m^−3^, and 0.61, respectively. In addition to these mean effects, the joint distribution of variables revealed partial overlap in responses between adjacent irrigation replacement levels. In particular, Si-treated plants receiving lower irrigation levels exhibited combinations of CWSI and yield similar to those observed in plants without Si receiving the next higher irrigation level, while generally maintaining higher WUE.

## 3. Discussion

Reduced irrigation progressively altered the coordination among plant water status and gas exchange. Across both tomato seasons, decreasing irrigation replacement reduced RWC, indicating a decline in the plant’s ability to maintain tissue hydration under restricted water availability. This response was accompanied by reductions in stomatal conductance and transpiration, reflecting a progressive restriction of gas exchange as water availability declined. Such regulation can reduce transpiration water loss and thereby contribute to the maintenance of plant water status. However, it also restricts CO_2_ diffusion into the leaf, which can constrain photosynthetic activity under water-limited conditions [30]. Accordingly, the simultaneous reductions in stomatal conductance, transpiration, and photosynthesis observed in the present study indicate that progressive water restriction affected both water conservation and carbon assimilation [2]. The decline in transpiration is particularly relevant because it represents not only a reduction in water loss but also a reduction in evaporative cooling. Thus, under increasingly restrictive irrigation regimes, the same stomatal response that limits water loss may also constrain the plant’s capacity to dissipate absorbed energy through transpiration, linking water conservation with thermal regulation [33].

The physiological restrictions imposed by reduced water availability were reflected in the canopy thermal response. As irrigation replacement decreased, the reduction in transpiration was accompanied by increases in canopy temperature, ΔT, and CWSI, reinforcing the connection between reduced evaporative cooling and canopy warming. Because canopy temperature results from the balance between absorbed radiation, latent heat loss through transpiration, sensible heat exchange, and surrounding atmospheric conditions, the observed increase in temperature under lower ETc levels indicates a shift in this energy balance as water availability became more restrictive [34,35]. The highest thermal stress values at 40% ETc are therefore consistent with the combined effects of reduced plant hydration, restricted gas exchange, and limited evaporative cooling, rather than representing an isolated thermal response to water deficit. Importantly, the same directional thermal gradient was observed across the two seasons despite differences in environmental conditions between seasons, supporting the interpretation that irrigation availability was a consistent determinant of the plant thermal response. Taken together, the concurrent changes in RWC, gas exchange, and canopy temperature indicate a progressive transition from a condition of greater physiological activity and evaporative cooling toward increasing water limitation. This integrated response also provides the physiological basis for interpreting the Si effects, particularly where Si application partially preserved transpiration and attenuated thermal responses under reduced irrigation [25].

Si application, using a concentrated liquid fertilizer containing 750 g L^−1^ of SiO_2_ applied via fertigation, was associated with improved physiological performance under the irrigation conditions evaluated. Across both seasons, plants with Si application showed increased photosynthesis, transpiration, and RWC, whereas its effect on stomatal conductance was significant only in the 2023–2024 season. This differential response is relevant because it indicates that the effects of Si were not expressed simply as a generalized increase in stomatal opening, but rather through a broader modulation of plant water relations and gas exchange [31,36]. These findings suggest that Si may influence stomatal dynamics indirectly by improving leaf water status rather than directly stimulating stomatal opening. The increase in RWC suggests that plants receiving Si were better able to maintain tissue hydration, while the greater transpiration indicates that this maintenance of water status was accompanied by continued transpirational activity [36]. Improved leaf hydration may contribute to maintaining stomatal function and transpirational cooling under water-limited conditions, thereby reducing leaf temperature. However, stomatal regulation involves complex physiological and biochemical processes, including hormonal signaling and changes in guard cell turgor [2,36].

Si application was associated with improved physiological performance under the irrigation conditions evaluated. Across both seasons, plants with Si application increased photosynthesis, transpiration, and RWC, whereas its effect on stomatal conductance was significant only in the 2023–2024 season. This differential response is relevant because it indicates that the effects of Si were not expressed simply as a generalized increase in stomatal opening, but rather through a broader modulation of plant water relations and gas exchange [31,36]. The increase in RWC suggests that plants receiving Si were better able to maintain tissue hydration, while the greater transpiration indicates that this maintenance of water status was accompanied by continued transpirational activity [36].

Under water-limited conditions, maintaining transpiration can be physiologically advantageous because it simultaneously reflects continued water movement through the plant and provides a mechanism for dissipating absorbed energy, contributing to leaf cooling [33]. Previous studies have associated Si application with improved plant water status and photosynthetic performance under water deficit, potentially through effects on water relations, stomatal regulation, membrane stability, and other physiological processes [23,37]. Taken together, these responses indicate that the physiological effect of Si involved a broader modulation of plant water relations and gas exchange, which was also reflected in canopy thermal behavior.

Canopy temperature integrates the effects of transpiration, radiation load, and the exchange of sensible and latent heat with the surrounding environment. Accordingly, the lower canopy temperature, ΔT, and CWSI observed with Si application indicate that these physiological responses were also expressed at the level of canopy energy balance [38]. This thermal modulation was particularly relevant under reduced irrigation, where the preservation of transpiration may have attenuated the thermal consequences of water limitation. Importantly, the lower thermal stress observed with Si does not indicate that water deficit was eliminated. Rather, it suggests that Si partially moderates the shift toward canopy warming as irrigation availability declined by maintaining transpiration and its associated heat dissipation [25,30].

In this context, the higher leaf Si accumulation observed under 80–100% ETc was accompanied by greater physiological activity and may reflect, at least in part, more favorable conditions for Si transport to aerial tissues through the transpiration stream [39]. However, the association between Si accumulation and transpiration should be interpreted cautiously, as Si uptake and deposition also depend on processes occurring at the root–shoot level [40]. Overall, the results indicate that the contribution of Si was expressed not by eliminating the physiological effects of water deficit, but by partially maintaining plant functioning and moderating its thermal consequences under reduced water availability.

Under 100% ETc, the relatively favorable water supply likely limited the scope for additional physiological benefits from Si, as plants were already operating under conditions of low water restriction. At the opposite extreme, the severe restriction imposed by 40% ETc likely became the predominant constraint, limiting the plant’s capacity to translate the potential benefits of Si into improvements in physiological and productive performance. The intermediate irrigation levels, particularly 60 and 80% ETc, represented a different physiological context in which water availability was sufficiently restricted to induce measurable stress while remaining adequate for the plants to retain responsiveness to Si. This pattern was supported by the significant ETc × Si interaction observed for transpiration and by the treatment-specific thermal responses. In 2022–2023, Si reduced CWSI from 0.80 to 0.50 at 60% ETc and from 0.44 to 0.33 at 80% ETc. Thus, the response pattern is better interpreted as context-dependent than as a linear increase or decrease in Si effectiveness with increasing water deficit. These results suggest that Si is most likely to express its potential when plants experience a degree of water limitation that is sufficient to generate stress but not so severe as to prevent the maintenance of physiological responsiveness [23,41].

The Hedges’ g estimates reinforce the effect-size pattern indicated that the magnitude of the Si response was generally greater under intermediate irrigation levels, with the strongest responses observed at 60% ETc for the variables evaluated. This finding is relevant because a statistically significant difference alone does not indicate whether the associated response is sufficiently large to be biologically or agronomically meaningful [42]. At 100% ETc, the relatively small effect sizes indicate limited additional benefit from Si under conditions in which water availability was already favorable. Whereas, under 40% ETc, the response to Si remained present but was less consistent across variables, suggesting that the more severe water restriction reduced the magnitude of some physiological and thermal responses to Si.

The intermediate levels therefore appear to define a range in which the crop retained sufficient functional capacity to respond to Si while experiencing enough water limitation for the effects of the bioinput to become evident. At 80% ETc, although the magnitude of the effects was lower than that observed at 60% ETc, thermal and gas-exchange variables still showed favorable responses to Si application. In contrast, under full irrigation or more severe water restriction, Hedges’ g estimates indicated a lower expression of the Si effect. Together, these results support the interpretation that Si effectiveness is conditioned by the intensity of water deficit, with its strongest overall expression occurring under a level of restriction that imposed measurable stress while still allowing physiological responsiveness.

A multivariate analysis of the observed responses showed that canopy temperature and CWSI were positioned in opposite directions to physiological and productive variables along the water restriction gradient, indicating that the thermal response accompanied coordinated changes in plant functioning under reduced water availability. Although this association does not establish a direct causal relationship among these responses, it indicates that thermal changes occurred concurrently with declines in plant water status, gas exchange, and yield. Physiologically, this pattern can be explained by the close relationship between transpiration and thermal regulation, as water limitation reduces latent heat loss through evaporation, thereby promoting increases in leaf temperature. This interpretation is consistent with Jackson et al. [34], who established canopy temperature as an indicator of crop water status, and Kibler et al. [33], who demonstrated the role of evapotranspiration in regulating leaf temperature.

Furthermore, previous studies have shown that indices derived from thermal imaging, such as CWSI, can be associated with plant water status and crop performance, reinforcing the potential of thermal information to reflect more than canopy warming alone [16]. Thus, in the present study, the thermal response not only followed the water restriction gradient but also reflected concurrent changes in physiological functioning and agronomic performance, reinforcing its value as an integrated indicator of plant condition under water deficit.

Regarding yield and water use efficiency, increasing irrigation replacement improved yield but reduced WUE, revealing a trade-off between water conservation and maintaining crop performance. This pattern is consistent with findings widely reported for deficit irrigation, in which reducing the amount of water applied tends to increase water use efficiency, although often at the expense of yield reductions [10]. In the present study, this contrast became more pronounced under the most restrictive irrigation levels. At 40% ETc, WUE reached 72.35 kg m^−3^ but was associated with a CWSI of 0.84 and a 33.7% reduction in yield compared with full irrigation. In contrast, 80% ETc resulted in a 20% reduction in applied water, a 14.7% increase in WUE, and only an 8.9% reduction in yield, indicating that similar objectives of improving water use efficiency may involve different physiological and productive costs.

This finding is particularly relevant because WUE represents the relationship between production and water use and may therefore increase even when yield declines, provided that the proportional reduction in water use exceeds the reduction in yield [43,44]. Thus, maximizing WUE alone does not necessarily represent the most agronomically favorable condition. Incorporating CWSI into the interpretation of this trade-off provides additional information on the physiological cost associated with water savings, as the greatest improvements in WUE coincided with higher thermal stress under the most restrictive irrigation treatments [44,45]. Therefore, the combined assessment of yield, WUE, and thermal response provides a more comprehensive basis for irrigation management decisions. Moreover, the lower CWSI values associated with Si application suggest that Si may partially shift this balance, favoring conditions in which reduced irrigation is accompanied by smaller physiological and productive penalties.

The results obtained demonstrate that the thermal response accompanies changes in plant water status, physiological functioning, and tomato yield, highlighting its potential to characterize plant condition under different levels of water availability. Building on this relationship, a relevant perspective is to investigate how thermal behavior develops throughout the crop cycle, allowing an assessment of whether thermal changes emerge before more pronounced physiological or productive responses. Studies employing plant thermal monitoring have highlighted the potential of these variables to track changes in plant water status and support the identification of stress progression over time [16]. In this context, repeated measurements of canopy temperature, ΔT, and CWSI throughout the crop cycle could reveal not only stress intensity but also the stage at which water restriction begins to substantially affect crop functioning [46].

Furthermore, such monitoring would make it possible to determine whether Si-associated thermal modulation emerges early and persists as water deficit progresses. Characterizing this temporal behavior would also allow investigation of whether thermal signals precede more pronounced physiological and yield losses. Therefore, an important step toward applying thermal imaging in irrigation management is to incorporate the temporal dimension of the thermal response, enabling earlier identification of stress progression and providing complementary information for decision-making before water restriction leads to more severe physiological and productive penalties.

## 4. Materials and Methods

### 4.1. Experimental Area

**Figure 8:**
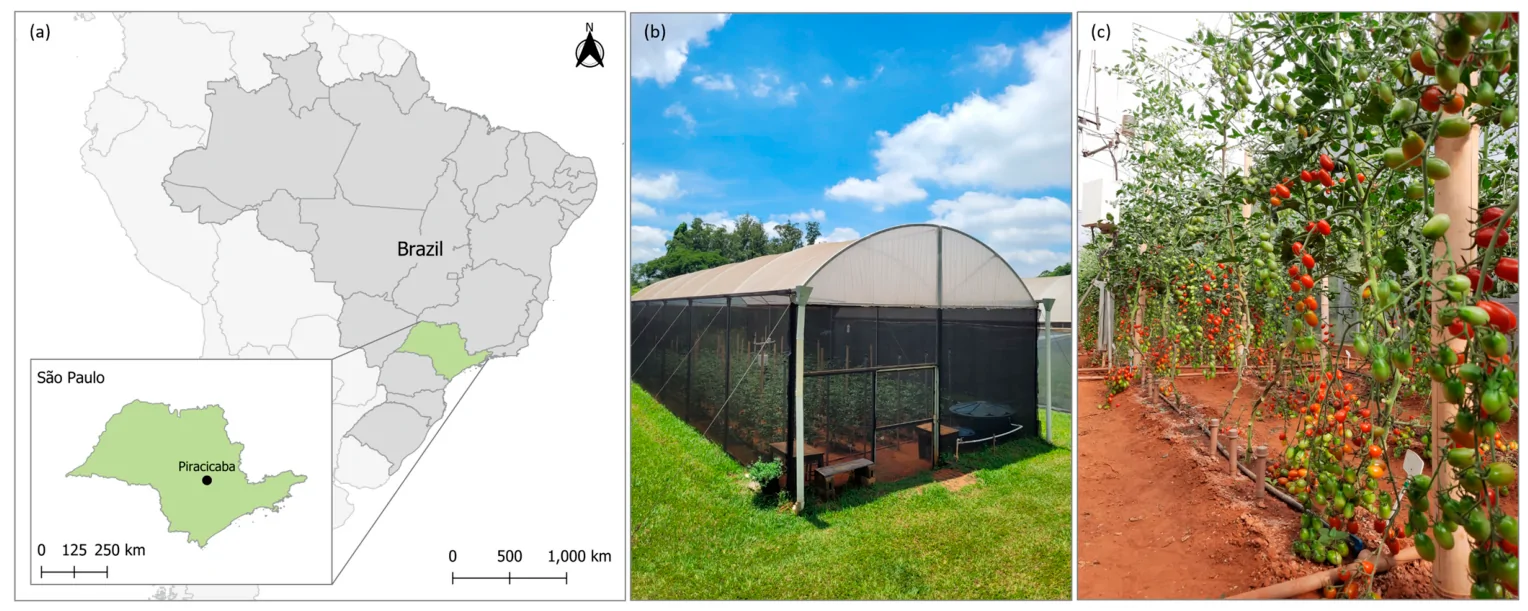
Location of the experimental area. (**a**) Location map of the experiment in the municipality of Piracicaba, São Paulo, Brazil; (**b**) external view of the protected environment used in the study; (**c**) internal view of the tomato crop. The map was generated by the authors using QGIS version 3.40.14 (https://qgis.org/download/), (accessed on 22 April 2026). State boundaries were obtained from the Brazilian Institute of Geography and Statistics (IBGE; https://www.ibge.gov.br/geociencias/organizacao-do-territorio/malhas-territoriais.html), (accessed on 22 April 2026) and country boundaries from the Database of Global Administrative Areas (GADM; https://un-spider.org/links-and-resources/data-sources/database-global-administrative-areas-gadm), (accessed on 22 April 2026).

The study was conducted at the experimental area of the Department of Biosystems Engineering, “Luiz de Queiroz” College of Agriculture (ESALQ), University of São Paulo (USP), in Piracicaba, São Paulo, Brazil (22°42′40″ S, 47°37′46″ W; 546 m of altitude). The climatic conditions of the study region are characterized by an annual mean air temperature of 21.6 °C, average relative humidity of 73%, and annual rainfall of approximately 1280 mm. According to the Köppen climate classification, the region is classified as Cwa, corresponding to a humid subtropical climate with hot, humid summers and dry winters [47]. The tomato plants were grown in a protected environment to ensure greater experimental control and data reliability while minimizing the influence of external climatic conditions. The experiment was conducted in a protected environment covering an area of 200 m^2^, with a 4.5 m eave height. The structure featured an arch-shaped roof covered with 150 µm-thick transparent light-diffusing plastic, while the sides were enclosed with high-density polyethylene anti-aphid nylon mesh (Figure 8).

The soil characterization is presented in Table 3. The soil of the experimental area is classified as a Red-Yellow Oxisol [48]. Before the beginning of the experiment, soil samples were collected from the 0–0.20 m and 0–0.40 m layer for physical, physical–hydraulic, and chemical characterization. Disturbed samples were used to determine the soil chemical properties [49], whereas undisturbed samples were collected for the assessment of physical and physical–hydraulic properties [50].

**Figure 9:**
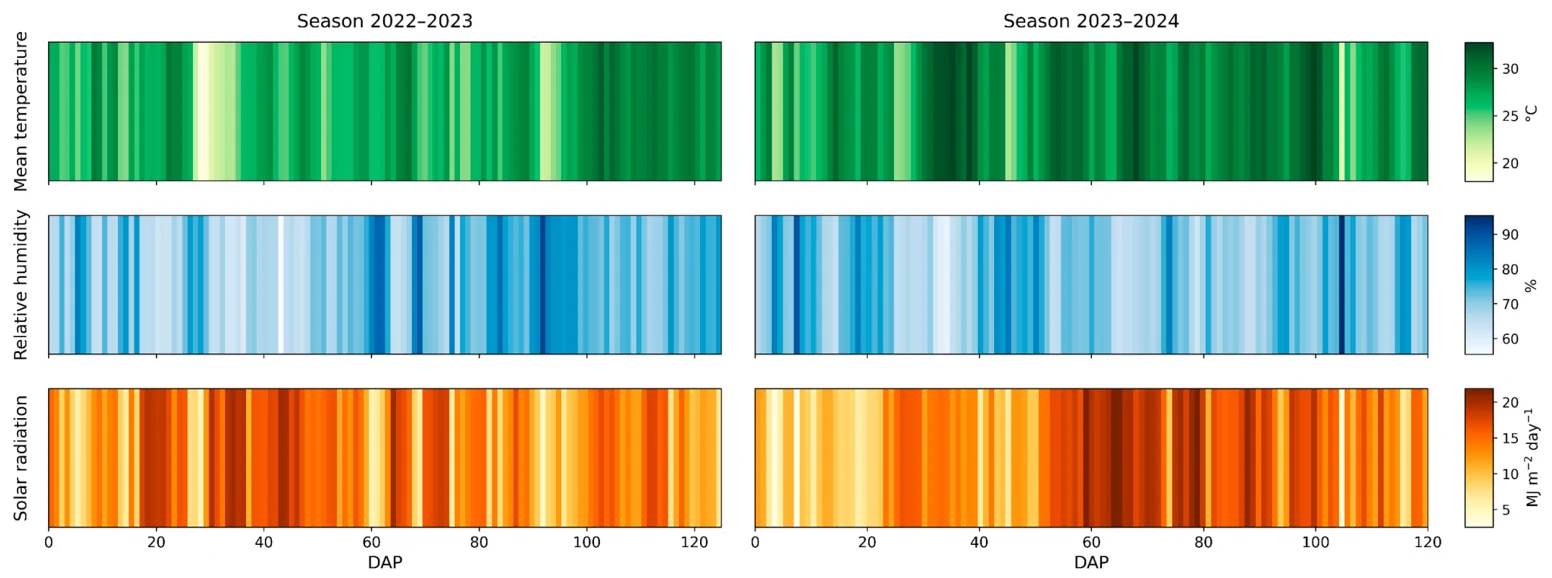
Climatic conditions recorded during the two tomato production cycles.

Meteorological conditions inside the protected environment were continuously monitored throughout the crop development. An automatic weather station was positioned at the center of the structure and equipped with sensors for air temperature and relative humidity (HMP45C), solar radiation (LI200X), and atmospheric pressure (CS106), all connected to a CR23X datalogger (Campbell Scientific, Logan, UT, USA). Measurements were recorded at 15 min intervals, and the daily maximum, minimum and average air temperature, average relative humidity, and the daily solar radiation values observed throughout both experimental crops cycles are presented in Figure 9.

### 4.2. Experiment Setup and Management

The study was conducted over two consecutive tomato growing cycles. The first cycle was carried out from 5 October 2022, to 7 February 2023, lasting 125 days, while the second cycle took place from 11 October 2023, to 8 February 2024, with a duration of 120 days. Before transplanting the seedlings, the soil was tilled and shaped into raised beds measuring 1.25 m in length, 0.40 m in width, and 0.20 m in height, corresponding to an effective area of 0.50 m^2^ per experimental unit. A 1 m spacing was maintained between beds to prevent water movement between experimental units during irrigation. At the time of bed preparation, 25 L of well-composted cattle manure were incorporated into each bed, corresponding to 336 kg ha^−1^ of N, 125 kg ha^−1^ of P_2_O_5_, 113 kg ha^−1^ of K_2_O, 136 kg ha^−1^ of Ca, and 42 kg ha^−1^ of Mg. Mineral fertilization was then applied to meet the nutrient requirements of tomato cultivation, using calcium nitrate, potassium nitrate, and single superphosphate, according to the recommendations of Trani et al. [51]. Three tomato plants were established in each experimental unit.

*Sweet Heaven*^®^, Bragança Paulista, Brazil, was selected as the tomato hybrid for this study. This grape-type mini tomato produces elongated, oblong fruits, with individual fruits weighing approximately 15 g on average. For seedling establishment, the seeds were initially germinated in polyethylene trays containing cells with a volume of 0.022 L, which were filled with a mixture of sphagnum peat and vermiculite. The seedlings remained in the nursery for 30 days following sowing. Once they had reached the appropriate transplanting stage, characterized by the presence of three to four true leaves, they were transferred from the trays and established in the soil of the experimental site.

Throughout the crop cycle, tomato plants were managed through a set of routine practices aimed at maintaining an appropriate canopy structure, supporting plant growth, and improving crop health. Because the hybrid exhibits indeterminate growth, the plants were vertically trained and supported using a staking system. A single-stem training system was adopted, with axillary shoots being periodically removed to avoid excessive vegetative growth and concentrate plant resources on reproductive development. Lower leaves were progressively eliminated as the fruits in the corresponding clusters advanced toward maturity, particularly when these leaves showed reduced physiological activity, which also contributed to improving air circulation within the canopy and reducing the risk of phytosanitary problems. Finally, 90 DAP the seedlings, the growing point was removed by apical pruning to restrict further vertical elongation and regulate plant development.

### 4.3. Experimental Design

The experiment was arranged in a randomized complete block design using a 4 × 2 factorial scheme, with four irrigation levels and two Si application conditions, resulting in 8 treatment combinations and four blocks, for a total of 32 experimental units. The irrigation treatments consisted of four fractions of crop evapotranspiration (ETc): 40, 60, 80, and 100 ETc. The 100% ETc treatment was adopted as the reference condition, representing the full replacement of crop water requirements. Irrigation levels of 40, 60, and 80% ETc were established to impose different degrees of water deficit, with 40% and 60% ETc representing severe water restriction and 80% ETc corresponding to a moderate deficit.

The second experimental factor comprised the application or non-application of Si. Totale Silício^®^, Santa Clara, Brazil, a highly concentrated liquid fertilizer containing soluble Si oxide (SiO_2_), was used as the Si source. The product contains 750 g L^−1^ of SiO_2_ and 45 g L^−1^ of K_2_O, with a density of 1.20 g mL^−1^, and was applied via fertigation at the recommended concentration of 1.25 mL L^−1^. The Si source also supplied potassium, corresponding to an additional 8 kg ha^−1^ of K_2_O, equivalent to approximately 3% of the total K_2_O supplied through mineral fertilization and cattle manure. Although this additional potassium input was exclusive to Si-treated plants, its relatively small contribution to the overall potassium supply suggests that it was unlikely to be the primary factor responsible for the observed responses. Si applications were performed at five stages of the crop cycle, corresponding to 15, 30, 45, 60, and 75 DAP. At each application, 2 L of the Si-containing solution were supplied to each experimental unit through the irrigation system. For treatments without Si, the same volume was applied with only irrigation water.

### 4.4. Irrigation System and Management

The Irrigation was supplied to the crop using a localized drip system, with one drip line installed along each experimental unit. The lines had an internal diameter of 0.016 m and incorporated seven pressure-compensating, anti-siphon emitters, positioned at 0.20 m intervals. Each emitter delivered 1.6 L h^−1^, resulting in a nominal discharge of 11.2 L h^−1^ per lateral line. The irrigation network was connected to a 0.025 m diameter PVC mainline and operated using a KSB 500N pump, which maintained a working pressure of approximately 1.0 bar. Individual irrigation control was provided for each experimental unit, allowing the application of the different irrigation treatments independently.

Prior to the beginning of the experiment, the performance of the drip irrigation system was assessed through the Christiansen Uniformity Coefficient (CUC). The calculated values were 98.8% and 98.2% for the first and second crop cycles, respectively, demonstrating a high degree of water distribution uniformity and confirming the suitability of the system for experimental use. These values were considered as the water application efficiency (Ea) of the irrigation system for each crop cycle. Irrigation water was obtained from the water treatment facility of the “Luiz de Queiroz” College of Agriculture. The water presented an apparent color below 0.2 Hazen units, electrical conductivity of 135.1 µS cm^−1^, pH of 7.0, turbidity of 0.4 turbidity units, total CaCO_3_ concentration of 45 mg L^−1^, calcium hardness of 32.2 mg L^−1^, and magnesium hardness of 13.5 mg L^−1^ indicating adequate water quality for irrigation and fertigation applications.

Irrigation scheduling was established based on crop evapotranspiration (ETc), which was used to determine the water depth required at each irrigation event (Equation (1)). The ETc was estimated according to the FAO-56 methodology, considering the atmospheric evaporative demand and the water requirements of the tomato crop at its different phenological stages. Reference evapotranspiration (ETo) was first calculated using the FAO-56 Penman–Monteith method (Equation (2)). The Kc values adopted were 0.50, 0.80, 1.25, 0.95, and 0.65 for the initial, development, mid-season, late-season, and harvest stages, respectively, based on Doorenbos and Kassam [52] and Allen et al. [53].

ETc = ETo Kc(1)

where ETc is the crop evapotranspiration (mm day^−1^), ETo is the reference evapotranspiration (mm day^−1^), and Kc is the crop coefficient (dimensionless).

(2) ETo=0.408ΔRn − G+γ(900T +273)  u2(es −ea)Δ+γ(1+0.34 u2)

where Rn is the surface radiation balance (MJ m^−2^ d^−1^), G is the soil heat flux density (MJ m^−2^ d^−1^), T is the air temperature at 2 m height (°C), u2 is the wind speed at 2 m height (m s^−1^), es is the saturation vapor pressure (kPa), ea is the partial vapor pressure (kPa), Δ is the slope of the saturation vapor pressure curve (kPa °C^−1^) and γ is the psychrometric constant (kPa °C^−1^).

At the beginning of the experiment, all experimental units were irrigated until field capacity was reached and this condition was maintained during the first 10 DAP to promote uniform seedling establishment. From 11 DAP onward, the experimental irrigation treatments were initiated. The 100% ETc treatment was considered the reference condition and received the full water depth estimated from the climate-based approach. The other irrigation treatments were established as proportional fractions of the 100% ETc level, corresponding to the different deficit and excess water regimes evaluated. The irrigation depth (ID) for each treatment was calculated using Equations (3)–(6) considering the Ea of 98.8 and 98.2% for the first and second crop cycles, respectively. Irrigation events were scheduled at two-day intervals throughout the crop cycle and carried out at 8:00 a.m., with the applied water depth adjusted according to the ETc estimated for each irrigation period.

(3) $${\mathrm{ID}}_{100}=\frac{\mathrm{ETc}}{\mathrm{Ea}}$$

ID_80_ = (ID_100_) 0.80 (4)

ID_60_ = (ID_100_) 0.60 (5)

ID_40_ = (ID_100_) 0.40 (6)

where ID_100_ is the irrigation depth for 100% ETc replacement (mm), ID_80_ is the irrigation depth for 80% ETc replacement (mm), ID_60_ is the irrigation depth for 60% ETc replacement (mm), ID_40_ is the irrigation depth for 40% ETc replacement (mm), ETc is the crop evapotranspiration (mm day^−1^), and Ea is the application efficiency (expressed as a decimal).

### 4.5. Thermal Image Data Acquisition and Processing

Canopy thermal measurements were obtained using a FLIR T640 Duo Pro R infrared camera, operating within the 7.5–13.5 µm spectral range. The camera provided thermal images with a spatial resolution of 640 × 512 pixels and a thermal sensitivity below 50 mK. Before each acquisition, the camera settings were adjusted according to the environmental conditions observed during the measurement, while the surface emissivity was fixed at 0.95. For each experimental unit, a single thermal image was collected between the mid-season and late-season phase of the tomato crop cycle, at 80 DAP. To ensure comparability among treatments, image acquisition was conducted under standardized conditions, with the camera positioned approximately 1.0 m above the crop canopy and oriented perpendicular to the plant surface. Images were recorded between 12:00 and 13:00 local time under clear-sky conditions and in the absence of cloud interference. The same acquisition protocol was maintained for all experimental units.

Thermal images were subsequently analyzed in FLIR Thermal Studio^®^ software (v. 2.0.11). Initially, each image was spatially cropped to exclude areas outside the corresponding experimental unit and reduce the influence of non-vegetative surfaces and background pixels. The thermal data were displayed using the “Iron” color palette, and identical minimum and maximum temperature limits were applied across images to facilitate comparisons among treatments. Canopy temperature was determined by defining a sampling transect along the central portion of the experimental unit, parallel to its longest axis. Five points were randomly selected along this transect, and the resulting temperature values were averaged to obtain a representative canopy temperature for each experimental unit.

Canopy temperature values were subsequently used to derive the thermal indicators, the normalized temperature difference (ΔT) and the Crop Water Stress Index (CWSI), which were employed to characterize the thermal response of tomato plants under the different irrigation and Si treatments. The ΔT was calculated using Equation (7). ΔT values can be negative, zero, or positive. A negative ΔT value indicates the absence of water stress, while a positive value corresponds to the presence of water stress [54].

ΔT = Tc − Ta(7)

where ΔT is the normalized temperature difference (°C), Tc is the canopy temperature (°C), and Ta is the air temperature at the time of Tc acquisition (°C).

To calculate the CWSI, two reference limits were required to normalize the effects of atmospheric conditions on canopy transpiration and temperature. These limits include the baseline for a non-stressed plant (Twet), representing a well-irrigated crop with no water restrictions, and the baseline for a water-stressed plant (Tdry), indicating a crop with limited or nearly no transpiration due to stomatal closure. The baselines were established based on real field conditions obtained from the experiments. Specifically, thermal image temperature thresholds from the 100% and 40% ETc irrigation treatments were used to represent Twet and Tdry, respectively. Therefore, the CWSI was calculated according to Jackson et al. [34] (Equation (8)). CWSI values can range from zero to one (0–1). A CWSI value of 0 indicates the absence of water stress, while a value of 1 corresponds to maximum water stress.

(8) $$\mathrm{CWSI}=\frac{\Delta T-\mathrm{Twet}}{\mathrm{Tdry}-\mathrm{Twet}}$$

where CWSI is the Crop Water Stress Index (dimensionless), ΔT is the normalized temperature difference (°C), Twet is the baseline canopy temperature of a non-stressed plant (°C), and Tdry is the baseline canopy temperature of a water-stressed plant (°C).

### 4.6. Gas Exchange and Plant Water Status

Physiological measurements were conducted at 80 DAP, coinciding with the same crop developmental stage at which the thermal image acquisitions were performed. Gas exchange parameters were measured using a LI-6400 XT infrared gas analyzer (LI-COR Biosciences, Lincoln, NE, USA). Measurements were performed on the fourth or fifth fully expanded leaf from the plant apex between 9:00 and 11:00 a.m., under clear-sky conditions and without cloud interference. The equipment was operated with a CO_2_ concentration of 400 μmol mol^−1^ and a photosynthetic photon flux density of 800 μmol m^−2^ s^−1^. The variables evaluated included net photosynthetic rate (μmol m^−2^ s^−1^), stomatal conductance (mol m^−2^ s^−1^), and transpiration rate (mmol m^−2^ s^−1^).

Plant water status was characterized through measurements of relative water content (RWC), which were assessed during the same phenological period as the gas exchange measurements. The RWC was determined on the fourth or fifth fully expanded leaf from the plant apex. Immediately after collection, the samples were weighed to determine fresh mass, subsequently immersed in deionized water for 24 h to obtain turgid mass, and then dried in a forced-air circulation oven at 65 °C for 72 h to determine dry mass. RWC was calculated from the relationship among fresh, turgid, and dry masses according to Equation (9) and expressed as a percentage.

(9) $$\mathrm{RWC}=\left(\frac{\mathrm{Fm}-\mathrm{Dm}}{\mathrm{Tm}-\mathrm{Dm}}\right)\times 100$$

where RWC is the relative water content (%), Fm is the fresh mass (g), Tm is the turgid mass (g) and Dm is the dry mass (g).

### 4.7. Yield and Water Use Efficiency

Harvesting was performed progressively as the fruits reached the appropriate maturity stage throughout the production cycle. After each harvest, the fruits from each experimental unit were weighed using a semi-analytical balance with a precision of 0.001 g. Tomato yield was determined based on the cumulative fresh mass of fruits harvested from each plant and expressed as kg plant^−1^.

Water use efficiency (WUE) was calculated to evaluate the relationship between tomato yield and water consumption under the different irrigation regimes (m^−3^), according to Equation (10), and expressed in kg m^−3^. This parameter was used to assess the capacity of the crop to convert the supplied irrigation water into fruit biomass.

(10) $$\mathrm{WUE}=\frac{\mathrm{Yield}}{\mathrm{IWA}}$$

where WUE is water use efficiency (kg m^−3^), yield express in (kg), and IWA is the water applied through irrigation (m^3^).

### 4.8. Silicon Concentration

To quantify Si content (g kg^−1^) in leaves and soil, spectrophotometric analysis was performed at 660 nm against a standard curve. Plant tissue samples were prepared using 1% NaOH as the extractant [49]. Soil extraction followed the methodology outlined by Korndörfer et al. [55] and Pereira et al. [56], employing a 0.01 mol L^−1^ CaCl_2_ solution.

### 4.9. Statistical Analysis

All data obtained were subjected to normality and homoscedasticity tests using the Shapiro–Wilk and Levene tests, respectively. Subsequently, an analysis of variance was performed at a 5% significance level with R Studio (R Project for Statistical Computing, v. 4.1.2). A mean comparison test (Tukey) was conducted after verifying significant interaction among the evaluated parameters for each crop cycle. Additionally, principal component analysis (PCA) was performed to summarize the multivariate variation in the dataset and identify the variables directions and their collinearity. PCA were calculated using plot-level observations from tomato seasons 2022–2023 e 2023–2024 reunidos, rather than treatment means. The magnitude of the silicon effect was estimated using Hedges’ g index [42], considering the pooled data from both growing seasons. It was calculated from the difference between the means of treatments with and without silicon, standardized by the pooled standard deviation and corrected for small sample sizes. The effect size was oriented according to the biologically desirable response. Effect sizes were classified by magnitude as low (0.50 ≤ |g| < 1.50), medium (1.50 ≤ |g| < 2.50), high (2.50 ≤ |g| < 3.50), and very high (|g| ≥ 3.50).

## 5. Conclusions

Under the conditions evaluated in this study, reduced irrigation progressively impaired tomato water status, gas exchange, and yield, accompanied by increases in canopy temperature, ΔT and CWSI. Si application helped attenuate some of these changes by contributing to the maintenance of RWC, photosynthetic activity, and transpiration, while reducing thermal stress. However, the magnitude of these responses varied according to the intensity of water restriction, with more pronounced physiological and thermal effects observed at 60% ETc and less consistent responses under severe water deficit. Regarding agronomic performance, irrigation at 80% ETc provided a favorable balance between water savings and yield, reducing applied irrigation water by 20% and increasing WUE by 14.7%, with an associated yield reduction of 8.9% compared with full irrigation.

The combined assessment of physiological, thermal, and productive variables indicated that canopy temperature and CWSI reflected changes in plant functioning under different levels of water availability. Although these associations do not establish direct causal relationships, the results suggest that thermal imaging and derived indices may complement conventional assessments of crop water status and physiological responses. In this context, integrating thermal information with yield and WUE has the potential to support the identification of irrigation strategies that balance water savings with the maintenance of agronomic performance. Nevertheless, the findings highlight the potential of thermal approaches to improve the understanding of integrated tomato responses to water restriction and Si application, contributing to a more comprehensive assessment of the physiological and productive consequences of water management strategies.

## Figures and Tables

**Table 1:** Physiological and hydric parameters of tomato plants produced under different irrigation replacement levels and silicon application.

Treatments	Photosynthesis	Stomatal Conductance	Transpiration	RWC	Photosynthesis	Stomatal Conductance	Transpiration	RWC
	μmol m^−2^ s ^−1^	mol m^−2^ s ^−1^	mmol m^−2^ s ^−1^	%	μmol m^−2^ s ^−1^	mol m^−2^ s ^−1^	mmol m^−2^ s ^−1^	%
ETc levels		Season 2022–2023				Season 2023–2024		
40%	24.25 c	0.16 c	7.57 d	73.20 c	24.95 d	0.16 d	7.42 c	71.68 d
60%	27.11 b	0.19 b	8.39 c	74.89 c	26.63 c	0.18 c	8.11 b	76.11 c
80%	28.48 b	0.21 a	8.79 b	79.17 b	29.22 b	0.22 b	8.68 a	80.39 b
100%	30.50 a	0.22 a	9.42 a	83.84 a	30.99 a	0.24 a	8.85 a	85.62 a
*p* value	<0.001	<0.001	<0.001	<0.001	<0.001	<0.001	<0.001	<0.001
Si								
−Si	26.86 b	0.19	8.35 b	76.59 b	27.21 b	0.19 b	7.62 b	77.29 b
+Si	28.31 a	0.20	8.74 a	78.96 a	28.69 a	0.21 a	8.91 a	79.61 a
*p* value	<0.001	ns	<0.001	<0.05	<0.001	<0.01	<0.001	<0.01
ETc × Si								
40% − Si	23.14	0.16	7.16 c	71.93	23.92	0.16	7.03 d	71.71
60% − Si	26.67	0.19	8.24 bc	72.80	25.63	0.17	7.34 d	75.12
80% − Si	27.64	0.21	8.56 b	78.51	27.85	0.22	8.00 b	79.22
100% − Si	29.99	0.22	9.42 a	83.14	30.43	0.24	8.11 b	83.10
40% + Si	25.36	0.17	7.98 c	74.48	25.98	0.16	7.81 c	71.66
60% + Si	27.55	0.19	8.54 b	76.99	27.63	0.20	8.88 ab	77.10
80% + Si	29.31	0.21	9.01 ab	79.83	29.58	0.22	9.35 a	81.55
100% + Si	31.02	0.23	9.43 a	84.54	31.55	0.25	9.58 a	88.13
*p* value	ns	ns	<0.05	ns	ns	ns	<0.01	ns

ETc: Crop evapotranspiration; Si: Silicon application. Different letters in the same production cycle and column are statistically different by Tukey test at 5% significance. ns: not significant. RWC: relative water content.

**Table 2:** Thermal parameters of tomato plants produced under different irrigation replacement levels and silicon application.

Treatments	Leaf Temperature	ΔT	CWSI	Leaf Temperature	ΔT	CWSI
	°C	°C	-	°C	°C	-
ETc levels	Season 2022–2023			Season 2023–2024		
40%	34.81 a	0.96 a	0.87 a	35.17 a	2.73 a	0.80 a
60%	33.74 b	−0.11 b	0.65 b	34.17 b	1.73 b	0.67 b
80%	32.57 c	−1.28 c	0.38 c	32.57 c	0.13 c	0.44 c
100%	31.47 d	−2.39 d	0.17 d	31.42 d	−1.02 d	0.31 d
*p* value	<0.001	<0.001	<0.001	<0.001	<0.001	<0.001
Si						
−Si	33.49 a	−0.35 a	0.60 a	33.75 a	1.31 a	0.62 a
+Si	32.80 b	−1.05 b	0.44 b	32.91 b	0.47 b	0.49 b
*p* value	<0.001	<0.001	<0.001	<0.01	<0.01	<0.001
ETc × Si						
40% − Si	34.94	1.09	0.89 a	35.83	3.39	0.89
60% − Si	34.40	0.55	0.80 a	34.58	2.14	0.73
80% − Si	32.83	−1.03	0.44 bc	33.26	0.82	0.54
100% − Si	31.80	−2.05	0.26 d	31.35	−1.09	0.33
40% + Si	34.68	0.82	0.86 a	34.51	2.07	0.72
60% + Si	33.08	−0.78	0.50 b	33.77	1.33	0.61
80% + Si	32.32	−1.53	0.33 c	31.87	−0.57	0.34
100% + Si	31.13	−2.72	0.08 e	31.49	−0.95	0.28
*p* value	ns	ns	< 0.05	ns	ns	ns

ETc: Crop evapotranspiration; Si: Silicon application. Different letters in the same production cycle and column are statistically different by Tukey test at 5% significance. ns: not significant. CWSI: crop water stress index.

**Table 3:** Soil characterization of the experimental area.

**Layer**	**pH**	**Ca**	**Mg**	**K**	**H**	**Al**	**CEC**	**P resin**	**S**	**Si**	**OC**
**m**	**-**	**mmolc dm^−3^**						**mg dm^−3^**		**g kg^−1^**	**g dm^−3^**
0–0.20	5.7	68.0	21.0	9.0	12.0	0.0	110.0	89.0	37.0	3.7	32.0
0.20–0.40	5.5	56.0	23.0	11.0	9.0	0.0	99.0	68.0	32.5	3.8	24.0
**Layer**	**B**	**Cu**	**Zn**	**Mn**	**Fe**	**Clay**	**Silt**	**Sand**	**ds**	**θ_FC_**	**θ_PWP_**
**m**	**mg dm^−3^**					**%.**			**g cm^−3^**	**m^3^ m^−3^**	
0–0.20	0.2	1.2	2.7	8.3	9.1	54.93	15.26	29.81	1.28	0.42	0.29
0.20–0.40	0.2	1.4	2.6	9.3	8.4	56.10	15.88	28.02	1.29	0.41	0.28

pH: hydrogen potential; Ca: calcium; Mg: magnesium; K; potassium; H: hydrogen; Al: aluminum; CEC: cation exchange capacity; V: base saturation; OC: organic carbon; P: phosphorus; S: sulfur; Si: silicon; B: boron; Cu: Copper; Zn: zinc; Mn: manganese; Fe: iron; ds: soil bulk density; θ_FC_: field capacity moisture; θ_PWP_: wilting point moisture.

## Data Availability

Data will be made available upon request.
